# Selection and Verification of Candidate Reference Genes for Mature MicroRNA Expression by Quantitative RT-PCR in the Tea Plant (*Camellia sinensis*)

**DOI:** 10.3390/genes7060025

**Published:** 2016-05-28

**Authors:** Hui Song, Xiao Zhang, Cong Shi, Shuangshuang Wang, Ailin Wu, Chaoling Wei

**Affiliations:** State Key Laboratory of Tea Plant Biology and Utilization, Anhui Agricultural University, 130 Changjiang West Road, Hefei 230036, China; 15155009532@163.com (H.S.); changkong718@163.com (X.Z.); sc104031227@163.com (C.S.); wangshuang0103@163.com (S.W.); wuailin19930331@163.com (A.W.)

**Keywords:** *Camellia sinensis*, microRNA, candidate reference gene, qRT-PCR, tea plant, expression

## Abstract

Quantitative reverse transcription-polymerase chain reaction (qRT-PCR) is a rapid and sensitive method for analyzing microRNA (miRNA) expression. However, accurate qRT-PCR results depend on the selection of reliable reference genes as internal positive controls. To date, few studies have identified reliable reference genes for differential expression analysis of miRNAs among tissues, and among experimental conditions in plants. In this study, three miRNAs and four non-coding small RNAs (ncRNA) were selected as reference candidates, and the stability of their expression was evaluated among different tissues and under different experimental conditions in the tea plant (*Camellia sinensis*) using the geNorm and NormFinder programs. It was shown that miR159a was the best single reference gene in the bud to the fifth leaf, 5S rRNA was the most suitable gene in different organs, miR6149 was the most stable gene when the leaves were attacked by *Ectropis oblique* and U4, miR5368n and miR159a were the best genes when the leaves were treated by methyl jasmonate (MeJA), salicylic acid (SA) and abscisic acid (ABA), respectively. Our results provide suitable reference genes for future investigations on miRNA functions in tea plants.

## 1. Introduction

MicroRNAs (miRNAs) are single-stranded, noncoding small RNAs, with a length of about 21 nucleotides (nt), which play critical regulatory roles involved in developmental processes and in response to biotic and abiotic stresses in plants [1,2,3]. Currently, miRNAs are attracting significant attention, and an increasing number of conserved and novel miRNAs have been identified in different plants [4,5]. Expression level analysis of miRNAs is particularly important in exploring their biological functions. Recent studies have focused on the quantification of the expression of miRNAs using the quantitative reverse transcription-polymerase chain reaction (qRT-PCR) with stem-loop primers [6,7,8]. qRT-PCR has recently been demonstrated to be a powerful tool for studying and quantifying gene expression. qRT-PCR is simpler to carry out, is more accurate, specific, sensitive, and reproducible, has a broader range of effect sizes that it can detect [9,10,11,12] and has been widely used to analyze the expression of genes among different tissues and across different experimental conditions in plants [13,14,15]. However, accurate and reliable analysis of gene expression using qRT-PCR depends critically on the selection of appropriate internal controls for normalization [16,17]. Such internal controls, or reference genes, should be expressed at a constant level across various experimental conditions, such as in tissues grown indifferent environments or exposed to different biotic or abiotic stresses [18,19]. Beta-actin, alpha-Tubulin, ubiquitin, 18S ribosomal RNA and glyceraldehyde 3-phosphate dehydrogenase have been frequently used as internal references in qRT-PCR analysis of the expression of genes [20,21,22,23], but these genes are not wholly appropriate as internal controls for the measurement of the expression of miRNA. Consequently, it is very important to identify suitable internal reference genes. To date, very few reference genes have been identified for the quantitative analysis of miRNA expression in plants; snoR14 has been shown to have high stability in somatic embryogenesis under temperature treatments and adult tissues in citrus [24] and miR156b and miR1520d have proved to have the highest stability of expression in soybean experiments [17]. Of importance, a stable reference gene in one plant may not be suitable for normalization of miRNA expression in others. Consequently, there is no recognized inner reference gene for normalization in different plants.

The tea plant (*Camellia sinensis*) is a globally important commercial plant, growing as a perennial woody shrub in warm and damp climates [25]. Tea is consumed by more than two thirds of the world’s population and has positive health effects [26]. Cultivation of tea occurs in many countries, but particularly China, Japan and Korea [27,28]. The normal growth of tea plants is slow, leaving plants vulnerable to numerous environmental and physiological stresses that may negatively affect growth, yield and quality. These include biotic attacks (e.g., insects) [29] and abiotic stress (e.g., cold) [30].There is currently a focus on the microRNAs of tea plants. Some conserved miRNAs have been predicted based on sequence homology with full length nucleotide sequence databases [31,32,33] and six novel miRNAs have been identified by isolating and cloning of small RNA libraries [34]. Recently, Jeyaraj *et al.* (2014) [35] analyzed expression and validation of several *Camellia sinensis* miRNAs in bud tissues by using the stem-loop pulse qRT-PCR method. A total of 18 and 14 conserved cold responsive miRNA families were identified from two cultivated varieties [33]. However, no published studies have reported on reference gene identification of miRNA qRT-PCR in tea plants.

In this study, the expression of seven candidate reference genes was analyzed across a set of 15 samples, including buds and leaves from the first to the fifth leaf in shoots, four organs (leaf, root, flower and fruit), leaves treated by *Ectropis oblique* feeding, mechanical wounds and phytohormones (MeJA, SA, ABA). The study aimed to identify suitable internal reference genes for normalization of miRNA qRT-PCR data from buds and leaves, with different leaf positions and different organs and under biotic and abiotic stresses. This study is the first report on the selection of reference genes for quantitative analysis expression of miRNA by qRT-PCR in the tea plant.

## 2. Materials and Methods

### 2.1. Plant Material and Experimental Stress Treatments

As described previously [29], plant samples were collected in the tea plantation located at Anhui Agricultural University, Hefei, China. Clone cuttings from two-year-old tea plants (*Camellia sinensis* cv. Shuchazao) were cultured in pots (30 cm diameter, 35 cm height) and grown in a controlled environment. All experimental and control treatments were carried out in triplicate, with all replicates for a given experiment being processed at the same time. Flowers, fruits, roots and young leaves were collected from an 8-year-old tea plant grown in the natural environment. The bud to the fifth leaf were collected from the uppermost leaf down to the fifth leaf on a single branch. Fresh leaf samples were collected at different developmental stages and different sites on the plants. All samples were immediately frozen in liquid nitrogen after picking and stored at −80 °C prior to RNA extraction.

For insect-feeding treatments, tea geometrids (*Ectropis oblique*) in the 3rd larval stage were placed on tea plants (20 geometrids per individual tea plant). The leaves were collected after 1/3 of each leaf had been consumed by the geometrids. For mechanical wounding, tea leaves were damaged by autoclaved scissors to remove a similar amount of leaf tissues as in the insect feeding treatment. Tea leaves of the same age and position on non-treated plants were used as controls. All treated and control leaves were collected at 24 h after treatments, immediately frozen in liquid nitrogen and stored at −80 °C prior to RNA extraction. 

For hormone treatments, clone cuttings from other young tea plants were selected and sprayed evenly with exogenous chemicals: methyl jasmonate (MeJA) (2.5 mM, containing 0.05% Tween-20), salicylic acid (SA) (1 mM), or abscisic acid (ABA) (100 µM) until the leaves were completely wet. Control cuttings were sprayed with a 0.05% Tween-20 water solution, or distilled water. Leaves were collected at 48 h after MeJA, SA, or ABA treatments, immediately frozen in liquid nitrogen and stored at −80 °C prior to RNA extraction. 

### 2.2. Selection of Candidate Reference Genes

In this study, seven candidate reference genes were selected in order to identify the most stably expressed reference gene(s) for use in the miRNA qRT-PCR studies. These genes were selected on the basis of results from (unpublished) high-throughput sequencing of *Camellia sinensis*, which provided information on small RNA (sRNA) abundance. Sequences of three miRNAs (miR6149, miR159a and miR5368n) were validated by microarray hybridization, while three small noncoding RNAs (snRNAs) (U6, U4 and 5S rRNA) were cloned and sequenced. In addition, the nucleotide sequence for *C. sinensis* 5.8S rRNA was obtained from GenBank (Accession HM061514.1) (Bethesda, MD, USA). PCR primers (listed in Table S1) were designed using Primer Premier version 5.0 (Premier Biosoft International, Palo Alto, CA, USA) [36], within conserved regions of nucleotide sequences obtained from GenBank and aligned by DNAMAN version 6.0 (Lynnon Biosoft, San Ramon, CA, USA). 

### 2.3. Primer Design for Reverse Transcription of ncRNAs

miRNA stem-loop primers used for miRNA cDNA synthesis were designed according to Chen *et al.* (2005) [37]. The stem-loop primer sequence consists of 44 conserved and six variable nucleotides that are specific to the 3′ end of the miRNA sequence (5’GTCGT ATCCAGTGCAGGGTCCGAGGTATTCGCACTGGATACGACNNNNNN3′). Forward primers were designed based on miRNA sequences; the reverse primer is universal. To amplify the other four ncRNAs, which have longer templates, qRT-PCR primers were designed using Primer Premier Version 5.0 (Premier Biosoft International, Palo Alto, CA, USA) [36]. For ncRNAs, the reverse primers for PCR were also used for reverse transcription. All primer sequences are given in Table 1.

### 2.4. RNA extraction, cDNA synthesisand qRT-PCR protocol for ncRNAs 

Total RNA was isolated from samples of tea plants using the miRcute miRNA isolation kit (Tiangen Biotech, Beijing, China), which is designed for purification of total RNA, including miRNA and other small RNA molecules (20–200 nt) in plants, following the manufacturer’s instructions. To avoid amplication from genomic DNA contamination, the isolated total RNA samples were treated with Buffer MZ supplied by the kit according to the protocol [38]. Three replicates of RNA isolation were conducted for each biological replicate. RNA concentration and purity were determined using a spectrophotometer (Nanodrop 2000; Thermo Fisher Scientific, Wilmington, DE, USA). Integrity of the RNA was verified by gel electrophoresis, first on an ethidium bromide-stained 2% agarose-TBE gel, then on a denaturing agarose-MOPS gel. Only the RNA samples with absorbance A260/A280 ratios between 1.8 and 2.1 and A260/A230 ratios higher than 2.0 were used for further analysis. 

In preparation for qRT-PCR, 100 ng total RNA was used to synthesize cDNA strands with the PrimerScript RT Enzyme (TaKaRa, Dalian, China) using the stem-loop primers (Table 1), and the pulse reverse transcription program was carried out [39]. 

To obtain proper cycle threshold (Cq) values for qRT-PCR, cDNAs of abundantly expressed 5S rRNA, 5.8S rRNA, U4 and U6 were diluted 100-fold before amplification, while cDNAs of moderately abundant expression of miR5368n, miR159a and miR6149 were used directly for amplification. Ten microliters of PCR mixture containing 5 µL SYBR^®^Premix Ex TaqTMII kit (TaKaRa, Dalian, China) and 0.5 µL cDNA template were loaded onto an CFX96 real time detection system (Bio-Rad, Hercules, USA) in a 96-well reaction plate, with thermocycling conditions as follows: 95 °C for 5 min, followed by 40 cycles of 95 °C for 5 s and 60 °C for 10 s. The specificity of the amplicons was verified for the presence of primer dimmers or non-specific amplicons by the presence of a single peak in the qRT-PCR melting curve products, and a single band with the expected size in a 2% agarose gel after electrophoresis. No-template controls were set to ensure no reagent contamination.

### 2.5. Evaluation of Expression Stability of Candidate Reference Genes

To visualize expression stability of the seven candidate reference genes, box plots of raw Cq values were produced for different tissues and stress conditions, respectively. For more sophisticated analysis, the expression stability was evaluated using geNorm [40] and NormFinder [41]. qRT-PCR data were averaged arithmetically in each case from three biological replicates, exported into an Excel spreadsheet (Microsoft Office 2010, Microsoft Corporation, Redmond, WA, USA), and Cq values converted according to the requirements of geNorm or NormFinder programs. GeNorm calculates a stability value, M, for a candidate reference gene compared to all other genes tested, defined as the average of the pairwise variation in expression for a given gene compared to each of the remaining tested genes. This is based on the principle that the expression ratio of an ideal reference gene to other ideal reference genes should remain constant in all samples and not be affected by experimental conditions [41,42,43]. Therefore, a lower M value suggests that gene expression is more stable. GeNorm software can also enable the exclusion of the most unstable gene and the recalculation of M, with the setting of a cutoff value [44]. Here, 1.5 was chosen as the cutoff value, based on previous work by Vandesompele *et al.* 2002 [40]. NormFinder, another algorithm for identifying the optimal normalization gene(s) among a set of candidates, uses an ANOVA-based model to consider intra- and inter-group variation in expression levels to calculate a stability value for gene expression [41].

### 2.6. Evaluation of Utility of the Candidate Reference Genes

To examine the expression stability of potential reference genes, the relative expression levels of three target miRNAs (miR172e, miR166d, miR319c) were measured under experimental conditions and normalized to the optimal reference gene pair and the least stable reference gene. The miRNA qRT-PCR amplification conditions were the same as those described above. The relative expression levels of the target genes were calculated according to the 2^−∆∆*C*T^ method [45]. Samples from non-stressed plants were used as control subgroups. A Student’s *t*-test was performed to compare pairwise differences in expression, with significance being (*p* < 0.05). Variance analysis was conducted on relative quantification results.

## 3. Results and Discussion

### 3.1. Verification of Amplification Specificity and Efficiency of the Primer Sets

The amplification efficiency for each primer set was determined in a qRT-PCR assay using a five-fold dilution series from a cDNA template. Primer efficiency is based on the template amplification doubling rate for a specific primer set during a PCR. When efficiency is 100%, it indicates that the cDNA target is duplicated in every PCR cycle during the exponential phase. Amplification products of the seven candidate reference genes from control samples of non-treated tea leaves showed that all primer pairs amplified a single band of the expected size, while no amplification was observed in no-template controls for each selected reference gene. These single bands were retrieved and sequenced. These resulting sequences perfectly matched or strongly resembled the templates (Figure S1). Specific amplification of the intended transcript was also confirmed by the appearance of a single peak in a dissociation curve analysis. Regression analysis of the primer pair showed that the coefficient of determination R^2^ was between 0.984 and 0.999 and the amplification efficiency (E) was between 96% and 126.5% (Figure S2) without non-specific amplification products (Figure S3).

### 3.2. Description of Expression Profiling of Candidate Reference Genes

**Figure 1:**
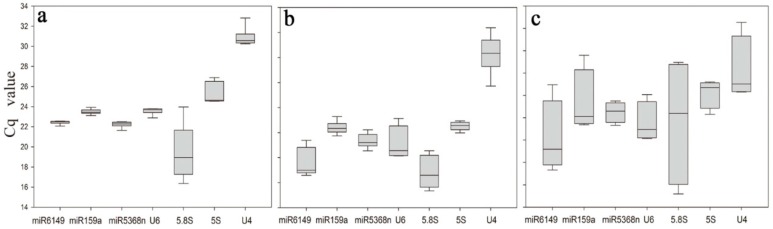
Box plots of the Cq values of different candidate reference genes among samples. (**a**) Expression data for seven candidate reference genes in tea plant leaves treated by *E. oblique* attack, mechanical wounding, MeJA, SA and ABA, respectively; (**b**) Cq values of seven candidate reference genes among six samples of the bud to the fifth leaf in tea plants; (**c**) Cq values (y-axis) of seven candidate reference genes of flowers, fruits, roots and young leaves in tea plants. Lines across boxes show the median, boxes show 25th and 75th percentiles and whisker caps show the maximum and minimum values and outliers.

Under the various experimental conditions, the expression levels of the seven candidate reference genes displayed quite different quantification cycle (Cq) values, spanning 16.37–30.82 (Figure 1a, Table S2). 5.8S rRNA was the least expressed gene with the lowest mean Cq value (16.37), and U4 had the highest mean Cq value (30.82). 5.8S rRNA showed the most variation in expression levels when both biological variation and experimental conditions were considered, as shown in Figure 1, even when amplified from a 100-fold dilution of cDNA reverse transcribed from 100 ng total RNA. Its lowest Cq value was under ABA treatment (16.37) and the highest Cq value was for the non-treatment sample (23.97), representing almost seven cycles of difference in Cq values. In contrast, miR6149 and miR159a were very stable candidate reference genes, with Cq values spanning 22.06–22.56 and 23.12–23.94, respectively, among different treatment samples. 

All seven candidate reference genes showed obvious differences in the bud to the fifth leaf tissue samples, with Cq values ranging from 19.35 to 32.38 (Figure 1b, Table S2). 5S rRNA, miR159a and miR5368n were relatively stable in their expression, with Cq values spanning 23.98–24.95, 23.74–25.29 and 22.54–24.22, respectively, while 5.8S rRNA was the most unstable, with Cq values of 19.35–22.55.

Differential expression was also present among different tissue samples, with Cq values spanning 17.8–32.54. Supplementary Table S2 includes also tissue samples (Figure 1c), which show quite large differences in Cq values between different tissues. This most probably indicates that more developed tissues are, the larger differences in Cq values exists in ncRNA expression (Figure 1c, Table S2). Expression of miR6149, miR159a and miR5368n was relatively stable among tissue samples, with Cq values spanning 19.32–26.96, 23.37–26.05 and 22.48–25.51, respectively, while 5.8S rRNA was again the most unstable, with Cq values spanning 17.8–28.97.

### 3.3. Expression Stability Analysis of All Candidate Reference Genes by GeNorm and NormFinder

**Figure 2:**
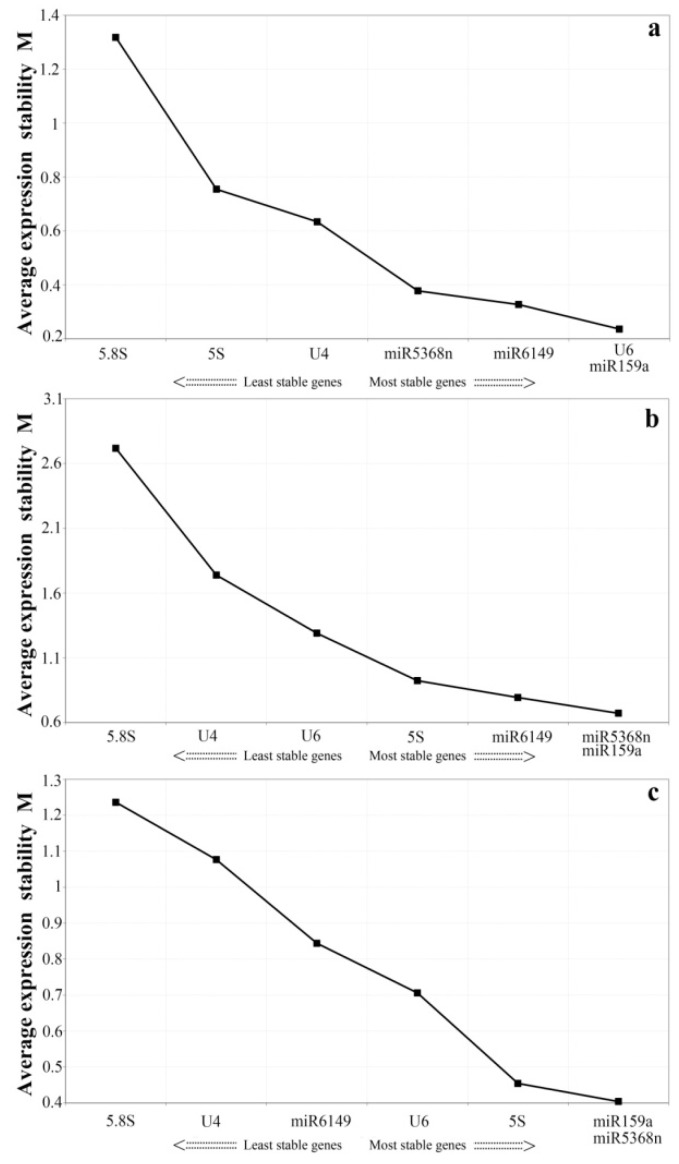
geNorm analysis of average expression stability values (M) and ranking of seven candidate reference genes. Average expression stability values (M) of candidate genes were measured during stepwise exclusion of the least stable reference genes. A lower value of average expression stability, M, indicates more stable expression. (**a**) Pooled samples from all treatments; (**b**) Pooled tissues; (**c**) In the bud to the fifth leaf.

In order to minimize bias, we selected the two different statistical analysis tools, geNorm and Normfinder, to calculate the expression stability of all the candidate reference genes. The geNorm program recommends the use of candidate reference genes with an average expression stability value, M, below the threshold of 1.5. A lower value of M shows more stable gene expression [40]. Candidate genes ranked by M value are shown in Figure 2. Here, all candidate genes showed stable expression in the two sample groups (the bud to the fifth leaf, and various treatments), each having an M value lower than 1.4 (Figure 2a,c). As shown in Figure 2b, in different tissues, the M values of U6，miR5368n, and 5S rRNA were lower than those of the other candidate genes, indicating that they are the most stably expressed genes according to geNorm, while miR159a, miR6149 ,U4 and 5.8S rRNA had an M value over 1.5 and are thus likely to be too untable to use as reference genes. For the samples of the bud to the fifth leaf, the three top-ranked candidates were miR159a, miR5368n and 5S rRNA. Under the biotic and abiotic stresses, the three most stably expressed genes were miR159a, miR6149 and U6.

NormFinder was used to perform an ANOVA-based analysis that takes intra- and inter-group variation into account for each candidate gene in evaluating expression stability [41]. Candidate genes with a lower average expression stability value are more stably expressed. The intra-group variation of each candidate gene is automatically calculated and converted into a stability value, which is shown, ranked, in Table 2. In the samples of the bud to the fifth leaf and of different organs, 5S rRNA, miR5368n and miR159a were the three top-ranked candidates, while in the pooled experimental stress treatments, U6, miR5368n and miR159a were the top-ranked genes. 5.8S rRNA was the least stable gene, congruent with the geNorm results.

### 3.4. Expression Stability Assessment of Candidate Reference Genes under Experimental Stresses and among the Bud to the Second Leaf

**Figure 3:**
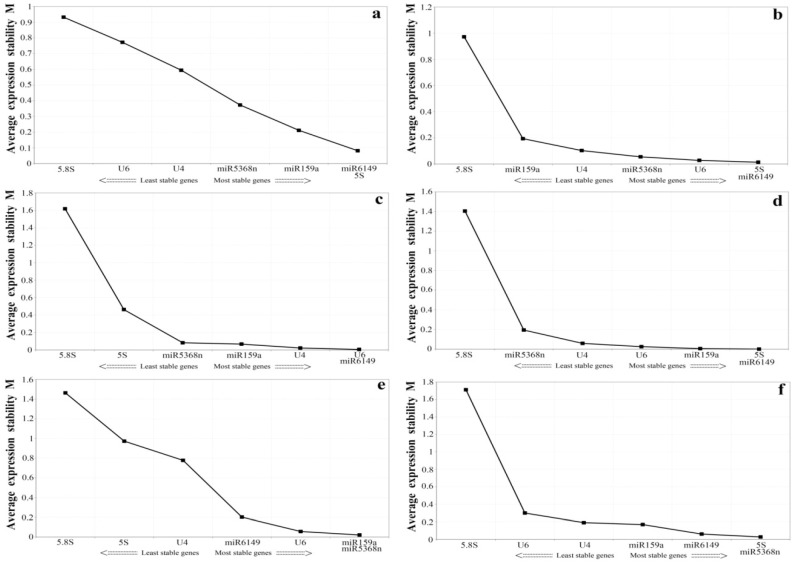
Average expression stability (M value) of seven candidate reference genes as calculated by geNorm. Expression stability was evaluated compared to untreated controls in (**a**) the bud to the second leaf; (**b**) *E. oblique* attack; (**c**) mechanical wounding; (**d**) SA; (**e**) MeJA; (**f**) ABA. A lower M value indicates more stable expression.

A separate analysis was conducted, evaluating individual stress and with samples of the bud to the second leaf. According to geNorm, the M values of the samples of the bud to the second leaf were lower than 1.0, with different conclusions on the most stable genes from the samples of the bud to the fifth leaf: miR159, miR5368n, miR6149 and 5S rRNA were the most stably expressed genes, while U6, U4 and 5.8S rRNA were ranked as the least stable genes (Figure 3a). When samples from insect feeding stress were analyzed using geNorm, the results were not the same as the pooled results from all biotic and abiotic stress conditions, but U6, miR6149 and 5S rRNA were the most stable genes(Figure 3b). Under mechanical wound conditions (Figure 3c), the three most stable genes were U6, U4 and miR6149. When SA was applied (Figure 3d), 5.8S rRNA and miR5368n ranked as the two least stable genes, while when MeJA was applied, the most stable were miR5368n, miR159a and U6 (Figure 3e). When ABA was applied, the three most stable candidate genes were miR5368n, miR6149 and 5S rRNA (Figure 3f).

Using NormFinder, when seven candidate genes were analyzed in the samples of the bud to the second leaf, miR6149 was the best reference gene, U6 was the most stable single gene under SA and mechanical wound treatments, while miR159a was most stable under MeJA and ABA treatments; miR5368n was one of the most stable genes under insect feeding treatment. As previously, 5.8S rRNA was the most variable gene when samples of the bud to the second leaf, and all individual stress conditions, were analyzed separately.

### 3.5. Determining the Minimum Number of Reference Genes Using GeNorm

**Figure 4:**
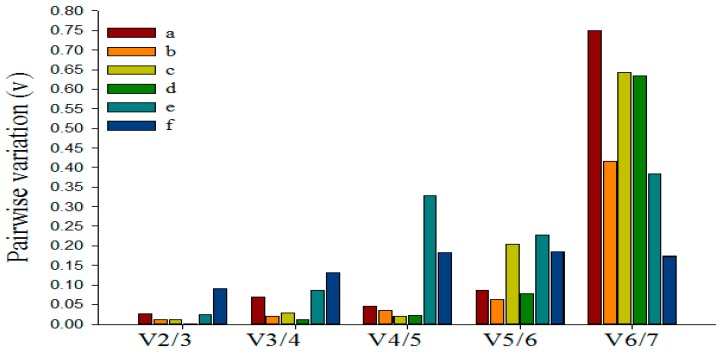
Determination of the optimal number of reference genes. Pairwise variation calculated by geNorm to determine the minimum number of reference genes for accurate normalization in: (**a**) *E. oblique* attack; (**b**) mechanical wounding; (**c**) SA; (**d**) MeJA; (**e**) ABA; (**f**) the bud to the second leaf. Each column shows a change in normalization accuracy when another endogenous control is added according to the ranking in Figure 3.

Previous researches have shown that using multiple reference genes as internal controls for normalization may generate more reliable results than using a single gene [40,46]. GeNorm software can be used to calculate the pairwise variation (V_n_/V_n+1_) between sequentially-added normalization factors to determine the optimal number of reference genes needed for accurate and reliable normalization [40]. The proposed cut-offvalue of V_n_/V_n+1_ is 0.15, below which the inclusion of an additional control gene is not required. As shown in Figure 4, for the six experimental samples, V_2_/_3_ valued 0.026, 0.012, 0.011, 0.002, 0.025 and 0.091, respectively, all of which were below the cut-off threshold of 0.15, demonstrating that the optimal reference pair in each group was the best choice of multiple reference genes (Figure 3). Therefore, we could consider miR6149 and 5S rRNA as the optimal multiple reference genes for the tea plant leaves treated by *Ectropis oblique* attack and SA, respectively, for the bud to the second leaf sample, U6 and miR6149 as optimal for the leaves damaged by mechanical wounding, and miR5368n and 5S as the best multiple reference genes for the leaves treated by MeJA, while miR5368n and miR159a were optimal for the leaves treated by ABA.

### 3.6. Evaluation of the Validity of a Selected Reference Gene Normalizing a Target Gene under Different Experimental Conditions

**Figure 5:**
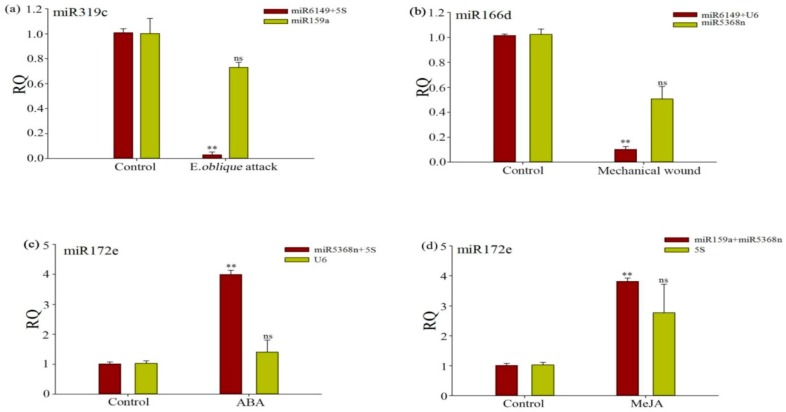
Evaluation of reference gene performance when normalizing target genes under experimental conditions. (**a**) Relative expression levels of miR319c in treated and non-treated plants, under *E. oblique* attack; (**b**) Relative expression levels of miR166d under mechanical wounding; (**c**,**d**) Relative expression levels of miR172e under ABA and MeJA application, respectively. Candidate reference genes used to normalize miR319c were miR6149 and 5SrRNA—the most stable reference genes under *E. oblique* attack, and miR159a—the unstable reference gene under *E. oblique* attack. Candidate reference genes used to normalize miR166d were miR6149 and U6—the most stable reference genes under mechanical wounding, and miR5368n—the most unstable reference gene under mechanical wounding. Candidate reference genes used to normalize miR172e were miR5368n and 5SrRNA—the most stable genes under ABA treatments, and U6—the most unstable gene under ABA treatments. Candidate reference genes used to normalize miR172e were miR5368n and miR159a—the most stable genes in MeJA treatments, and 5SrRNA—the most unstable gene under MeJA treatments. Asterisks (**) indicate very significant difference between the treated and non-treated plants, and no significance (ns) indicates no significant difference, based on a *t*-test (*p* < 0.01).

To demonstrate the reliability of the selected reference genes in qRT-PCR, the relative expression profiles of three target genes, miR319c, miR166d and miR172e were measured and normalized, using a single unstable reference gene and the optimal reference pair, as chosen using geNorm and NormFinder. Results were shown in Table 1 and Figure 3. As shown in Figure 5a, miR319c was very significantly induced (*p* < 0.01) under *E. oblique* attack using 5S + miR6149 as the optimal reference gene pair, but not when using miR159a as an unstable reference gene, with genes being chosen by NormFinder ranking (Table 3). miR166d was very significantly induced (*p* < 0.01) under mechanical wound conditions when U6 and miR6149 were used as the optimal reference pair (Figure 5b), but not when the single unstable reference gene (miR5368n) was used. However, miR166d expression did not increase and its standard deviation increased sharply under use of a single unstable reference gene. As shown in Figure 5c,d, miR172e was very significantly (*p* < 0.01) induced under ABA and MeJA treatment, when normalized by U6 as a single unstable reference gene, 5S a single unstable reference gene, and miR5368n + 5S as the optimal reference pair and miR5368n + miR159a as the optimal reference pair respectively. These results further confirmed the importance of the validation of reference genes when no single reference gene was observed to have constant expression across all the experimental conditions.

## 4. Conclusions

In this study, seven candidate reference genes, 5S rRNA, 5.8S rRNA, U4, U6, miR6149, miR159a and miR5368n were evaluated for the normalization of gene expression under different tissue and various stress conditions in the tea plant. Our results showed that miR6149, miR5368n, 5S rRNA and U6 were suitable as internal control reference genes, while 5.8S rRNA was clearly regulated differently under different tissue and various stress conditions, and was considered unsuitable as an internal control gene. Thus, appropriate combinations of miR6149, miR5368n, 5S rRNA and U6 were suggested as potential control genes for miRNA qRT-PCR normalization under different tissue and stress conditions. The validated reference genes of the present study should be useful in future quantitative RT-PCR studies of miRNA expression in the tea plant.

## Figures and Tables

**Table 1:** Templates and primers for qRT-PCR.

ncRNA	Template/Primer Sequences
miRNA6149	Seq: ATACGCACCTGAATCGGTAG RT: GTCGTATCCAGTGCAGGGTCCGAGGTATTCGCACTGGAT ACGACCTACCG F:GCGCGATACGCACCTGAAT
miRNA159a	Seq: CTTGGATTGAAGGGAGCTCC RT: GTCGTATCCAGTGCAGGGTCCGAGGTATTCGCACTGGATA CGACGGAGCT F: CGCGCTTGGATTGAAGGG
miRNA5368n	Seq: GAGATACCACTCTGGAAGAGC RT: GTCGTATCCAGTGCAGGGTCCGAGGTATTCGCACTGGAT ACGACGCTCTT F: GCGGAGATACCACTCTGG
miRNA319c	Seq: TTGGACTGAAGGGAGCTCC RT: GTCGTATCCAGTGCAGGGTCCGAGGTATTCGCACTGGAT ACGACGGAGCT F: GCGCTTGGACTGAAGGG
miRNA166d	Seq: TCGGACCAGGCTTCATTCCCCAG RT: GTCGTATCCAGTGCAGGGTCCGAGGTATTCGCACTGGAT ACGACCTGGGG F: GCTCGGACCAGGCTTCATT
miRNA172e	Seq: GGAATCTTGATGATGCTGCAT RT: GTCGTATCCAGTGCAGGGTCCGAGGTATTCGCACTGGAT ACGACATGCAG F: GCGCGGGAATCTTGATGATG
Universal reverse primer for miRNA qRT-PCR & reverse transcription	R: GTGCAGGGTCCGAGGTATTC
U6	Seq:GTCCCTTCGGGGACATCCGATAAAATTGGAACGATACAGAGAAGATTAGCATGGCCCCTGCGCAAGGATGACACGCACAAATCGAGAAATGGTCCAAATTTTTTT F: CGGGGACATCCGATAAAATTG R: GGACCATTTCTCGATTTGTGC
U4	Seq:GCAATGACGCAGCTAGTGAGGTAATAACCGAGGCGCGTCAATTGCTGGTTGAAAACTATTTCCAAACCCCCTCATTGGCCTGGGTTCAGCCCGGGCCTCTGAGAATTTCTGGAAGGGCTCCCTTTG F: TGAGGTAATAACCGAGGCGC R: CAGAAATTCTCAGAGGCCCG
5S -rRNA	Seq:CGGATGCGATCATACCAGCACCAATGCACTGGATCCCATCAGAACTATGCAGTTAAGCATGCTTGGGAGAGAATAGAGCTGGGGTGGGTGACCCCCTAGGAAGTCCTTGTGTTG F: GCGATCATACCAGCACCAATG R: CAACACAAGGACTTCCTAGGG
5.8S-rRNA	Seq:TAAACGACTCTCGGCAACGGATATCTCGGCTCTCGCATCGATGAAGAACGTAGCGAAATGCGATACTTGGTGTGAATTGCAGAATCCCGCGAACCATCGAGTCTTTGAACGCAAGTTGCGCCCGAAGCCATTAGGTTGAGGGCACGCCTGCCTGGGCGTCTCAC F: CTCGGCAACGGATATCTCG R: GCCCTCAACCTAATGGCTTC

Seq: template sequence; RT: stem-loop primer; F: forward primer for qRT-PCR; R: reverse primer for qRT-PCR.

**Table 2:** Ranking of candidate reference genes in order of normalization and their expression stability calculated using NormFinder.

Rank	The Bud to theFifth Leaf		Tissues		All Treatment Samples	
	Gene	Stability	Gene	Stability	Gene	Stability
1	5SrRNA	0.091	5SrRNA	0.4	U6	0.099
2	miR5368n	0.312	miR5368n	0.465	miR159a	0.193
3	miR159a	0.379	miR159a	0.525	miR5368n	0.211
4	U6	0.48	U6	0.574	miR6149	0.261
5	U4	0.579	U4	0.728	U4	0.341
6	miR6149	0.721	miR6149	0.921	5SrRNA	0.403
7	5.8SrRNA	0.835	5.8SrRNA	2.789	5.8SrRNA	1.019

Stability values are listed from the most stable to the least stable ones.

**Table 3:** Expression stability ranking of candidate reference genes as calculated by NormFinder.

Rank	The Bud to the Second Leaf		*E. oblique* Attack		Mechanical Wounding		SA		MeJA		ABA	
	Gene	Stab.	Gene	Stab.	Gene	Stab.	Gene	Stab.	Gene	Stab.	Gene	Stab.
1	miR6149	0.028	miR5368n	0.002	U6	0.002	U6	0.02	miR159a	0.007	miR159a	0.005
2	5S rRNA	0.028	miR6149	0.002	U4	0.002	miR5368n	0.123	miR5368n	0.007	U4	0.005
3	miR159a	0.113	5S rRNA	0.005	miR6149	0.002	miR6149	0.179	U6	0.022	U6	0.118
4	miR5368n	0.404	U4	0.051	miR159a	0.01	5S rRNA	0.179	miR6149	0.108	miR5368n	0.217
5	U4	0.684	U6	0.064	miR5368n	0.01	miR159a	0.194	U4	1.14	miR6149	0.379
6	U6	0.744	miR159a	0.452	5S rRNA	1.207	U4	0.339	5S rRNA	1.181	5S rRNA	0.461
7	5.8S rRNA	0.79	5.8S rRNA	2.025	5.8S rRNA	3.093	5.8S rRNA	3.085	5.8S rRNA	1.801	5.8S rRNA	3.626

Stability values are listed from the most stable to the least stable ones.

## Supplementary Materials

The following are available online at http://www.mdpi.com/2073-4425/7/6/25/s1. Figure S1: Alignments of qRT-PCR products to the corresponding ncRNA templates, Figure S2: E, qRT-PCR efficiencies, E = 10[–1/slope]; R2, coefficient of determination, Figure S3: Melting curves of ten genes of qRT-PCR, Table S1: Template and primer sequences for the three small noncoding RNAs (U6, U4, 5S rRNA) that were cloned, based on results from high-throughput sequencing by qRT-PCR, Table S2: Average Cq valueof candidate reference genes in the tested samples.

## Abbreviations

5.8S	5.8S ribosomal RNA
5S	5S ribosomal RNA
U6	U6 small nucleolar RNA
U4	U4 small nucleolar RNA
ABA	Abscisic acid
miRNA	microRNA
ncRNA	non-coding RNA
MeJA	Methyl Jasmonate
qRT-PCR	quantitative reverse transcription-polymerase chain reaction
