# Supplementary material for: Selection and Verification of Candidate Reference Genes for Mature MicroRNA Expression by Quantitative RT-PCR in the Tea Plant (Camellia sinensis)
Source: Genes (Basel). 2016 May 28;7(6):25. doi: 10.3390/genes7060025 (PMC4929424; doi:10.3390/genes7060025)
Supplement: Supplementary file 1 [file genes-07-00025-s001.pdf]

Table S1. Template and primer sequences for the three small noncoding RNAs (U6, U4, 5S rRNA) that were cloned, based on results from high-throughput sequencing by qRT-PCR.

ncRNA	Template/primer sequences(5′-3′)	Product Tm (℃)	Amplicon lenght
U6	F:TAAACGACTCTCGGCAAC	59.5	105
	R: GTGCAGGGTCCGAGGTATTC	62.5	
	RT:GTCGTATCCAGTGCAGGGTCCGAGGTATTCGCACTGGATACGACCAACAC		
5S-rRNA	F:TAAACGACTCTCGGCAAC	61.2	114
	R: GTGCAGGGTCCGAGGTATTC	62.5	
	RT:GTCGTATCCAGTGCAGGGTCCGAGGTATTCGCACTGGATACGACCAACAC		
U4	F: CTTTGCGCTTGGGGC	60.5	126
	R: CAAAGGGAGCCCTTCC	60.5	
	RT:GTCGTATCCAGTGCAGGGTCCGAGGTATTCGCACTGGATACGACAGGGCT		

Table S2. Average Cq value of candidate reference genes in the tested samples (mean ± SD; *n* = 3).

**a. Stress Treatments**	**5.8S rRNA**	**5S rRNA**	**U4**	**U6**	**miRNA6149**	**miRNA159a**	**miRNA5368n**
Control	23.97 ± 0.0208	24.57 ± 0.2899	30.57 ± 0.3512	23.68 ± 0.4071	22.5 ± 2.2155	23.42 ± 1.8644	22.29 ± 0.0751
E. oblique attack	19.92 ± 1.8967	24.61 ± 1.2562	30.36 ± 1.9463	23.78 ± 0.1537	22.56 ± 0.843	23.94 ± 0.5514	22.24 ± 0.3988
Mechanical wound	17.98 ± 0.0737	26.39 ± 0.1212	30.56 ± 0.1724	23.72 ± 0.1012	22.53 ± 0.9732	23.6 ± 0.4951	22.5 ± 1.1615
ABA	16.37 ± 0.9413	24.68 ± 0.3188	30.25 ± 0.3974	22.88 ± 0.7827	22.52 ± 0.0757	23.12 ± 0.1464	22.44 ± 1.3869
MeJA	20.88 ± 1.3952	26.89 ± 0.6298	32.82 ± 0.1804	23.8 ± 1.2396	22.06 ± 0.4887	23.45 ± 0.5877	22.29 ± 0.7885
SA	17.59 ± 0.129	24.55 ± 1.7087	30.69 ± 0.3477	23.6 ± 0.5353	22.48 ± 1.9168	23.41 ± 3.1871	21.63 ± 0.0889
**b. Bud + 5 leaves**	**miRNA6149**	**miRNA159a**	**miRNA5368n**	**U6**	**5.8S rRNA**	**5S rRNA**	**U4**
Single bud	20.59 ± 0.167	23.74 ± 0.164	22.54 ± 0.143	22.14 ± 0.167	22.55 ± 0.472	23.98 ± 0.115	30.46 ± 0.244
First leaf	20.94 ± 0.042	24.4 ± 0.248	23.32 ± 0.044	22.16 ± 0.105	20.62 ± 0.18	24.31 ± 0.113	31.07 ± 0.434
Second leaf	21.04 ± 0.219	24.54 ± 0.283	24.22 ± 0.117	24.33 ± 0.131	22.06 ± 0.067	24.56 ± 0.352	29.81 ± 0.378
Third leaf	22.63 ± 0.162	24.14 ± 0.104	23.08 ± 0.063	22.34 ± 0.204	19.35 ± 0.113	24.57 ± 0.175	27.72 ± 0.248
Fourth leaf	20.84 ± 2.297	24.27 ± 0.273	23.07 ± 0.321	22.78 ± 0.421	19.73 ± 2.754	24.94 ± 0.099	30.24 ± 0.147
Fifth leaf	23.38 ± 0.104	25.29 ± 0.19	23.73 ± 0.028	25.13 ± 0.183	20.57 ± 0.195	24.78 ± 0.501	32.38 ± 1.658
**c. Tissues**	**U6**	**U4**	**5.8S rRNA**	**5S rRNA**	**miRNA6149**	**miRNA159a**	**miRNA5368n**
Flower	23.52 ± 0.182	26.32 ± 0.046	17.8 ± 0.128	26.5 ± 0.039	21.16 ± 0.047	23.37 ± 0.031	25.18 ± 0.112
Fruit	26.08 ± 0.111	32.54 ± 0.089	28.05 ± 0.968	27.21 ± 1.888	21.21 ± 0.04	23.84 ± 0.183	24.81 ± 0.034
Root	22.37 ± 0.206	27.63 ± 0.41	28.97 ± 0.819	26.9 ± 0.307	26.96 ± 0.083	25.66 ± 0.122	25.51 ± 0.067
Leaf	23.45 ± 0.148	30.23 ± 0.182	23.95 ± 0.083	25.08 ± 0.302	19.32 ± 0.102	26.05 ± 0.22	22.48 ± 0.191

```
miR159a:               ----CTTGGATTGAAGGGAGCTCC---------------------------------
miR159a-PCR_product: CGCGCTTGGATTGAAGGGAGCTCCGTGTATCCAGTGCGAATACCTCGGACCCTGCAC
                           ********************

miR6149:               ----ATACGCACCTGAATCGGTAG----------------------------------
miR6149-PCR_product:  GCGCGATACGCACCTGAATCGGTAGGTCGTATCCAGTGCGAATACCTCGGACCCTGCAC
                            ********************

MiR5368n:              ---GAGATACCACTCTGGAAGAGC---------------------------------
miR5368n-PCR_product: GCGGAGATACCACTCTGGAAGAGCGTCGTATCCAGTGCGAATACCTCGGACCCTGCAC
                          *********************

miR166d:               --TCGGACCAGGCTTCATTCCCCAG--------------------------------
miR166d-PCR_product:  GCTCGGACCAGGCTTCATTCCCCAGTGTATCCAGTGCGAATACCTCGGACCCTGCAC
                          ***********************

miR319c:                ----TTGGACTGAAGGGAGCTCC----------------------------------
miR319c-PCR_product:   GCGCTTGGACTGAAGGGAGCTCCAGTGTATCCAGTGCGAATACCTCGGACCCTGCAC
                            *******************
miR172e:               -----GGAATCTTGATGATGCTGCAT-------------------------------
miR172e-PCR_product:  GCGCGGGAATCTTGATGATGCTGCATGTATCCAGTGCGAATACCTCGGACCCTGCAC
                           *********************

Csn-U6:              GTCCCTTCGGGGACATCCGATAAAATTGGAACGATACAGAGAAGATTAGCATGGCCCCTGCG-CAAGGATGACACGCACAAATCGAGAAATGGTCCAAATTTTTTT
Csn-U6-PCR_product:  -------CGGGGACATCCGATAAAATTGGAACGATACAGAGAAGATTAGCATGGCCCCTGCATCAAGGATGACACGCACAAATCGAGAAATGGTCC---------
                            ******************************************************. *********************************

Csn-5S_rRNA:          CGGATGCGATCATACCAGCACCAATGCACTGGATCCCATCAGAACTATGCAGTTAAGCATGCTTGGGAGAGAATAGAGCTGGGGTGGGTGACCCCCTAGGAAGTCCTTGTGTTG
Csn-5S-PCR_product:   ----GAGATCATACCAGCACTAATGCACCGGATCCCATCAGAACTCCGCAGTTAAGCGTGCTTGGGCGAGAGTAGTACTAGGATGGGTGACCCCCTGGGAAGTCCTCGTGTTG
                          *.************** ******* ****************. **********.********.****.***:.**.**.********************* ******
Csn-5.8S_rRNA:
 TAAACGACTCTCGGCAACGGATATCTCGGCTCTCGCATCGATGAAGAACGTAGCGAAATGCGATACTTGGTGTGAATTGCAGAATCCCGCGAACCATCGAGTCTTTGAACGCAAGTTGCGCCCGAAGCCATTAGGTTGAGGG

 CACGCCTGCCTGGGCGTCTCAC
Csn-5.8S-PCR product:
   ------CTCGGCAACGGATATCTCGGCTCTCGCATCGATGAAGAACGTAGCGAAATGCGATACTTGGTGTGAATTGCAGAATCCCGCGAACCATCGAGTCTTTGAACGCAAGTTGCGCCCGAAGCCATTAGGTTGAGGGC-

         **************************************************************************************************************************************
Csn-U4:              GCAATGACGCAGCTAGTGAGGTAATAACCGAGGCGCGTCAATTGCTGGTTGAAAACTATTTCCAAACCCCCTCATTGGCCTGGGTTCAGCCCGGGCCTCTGAGAATTTCTGGAAGGGCTCCCTTTG
Csn-U4-PCR_product: ----------------TGAGGTAATAACCGAGGCGCGTCAATTGCTGGTTGAAAACTATTTCCAAACCCCCTCATTGGCCTGGGTTCAGCCCGGGCCTCTGAGAATTTCTG-------------
                                *******************************************************************************************
```

Figure S1. Alignments of qRT-PCR products to the corresponding ncRNA templates.

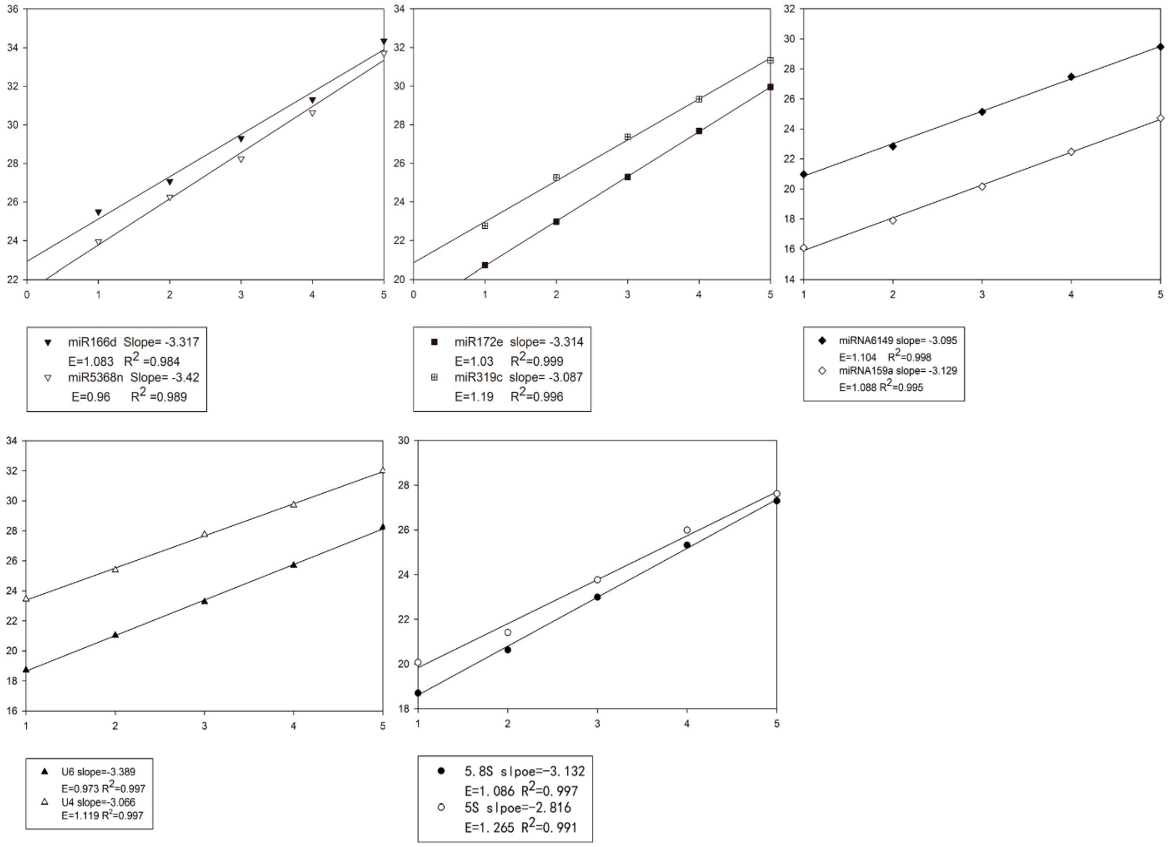

Figure S2. E, qRT-PCR efficiencies, E = 10[−1/slope]; R2, coefficient of determination; X axis: Cq, cycle threshold value (mean ± SD; *n* = 3); Y axis: logarithmic dilution fold of cDNA.

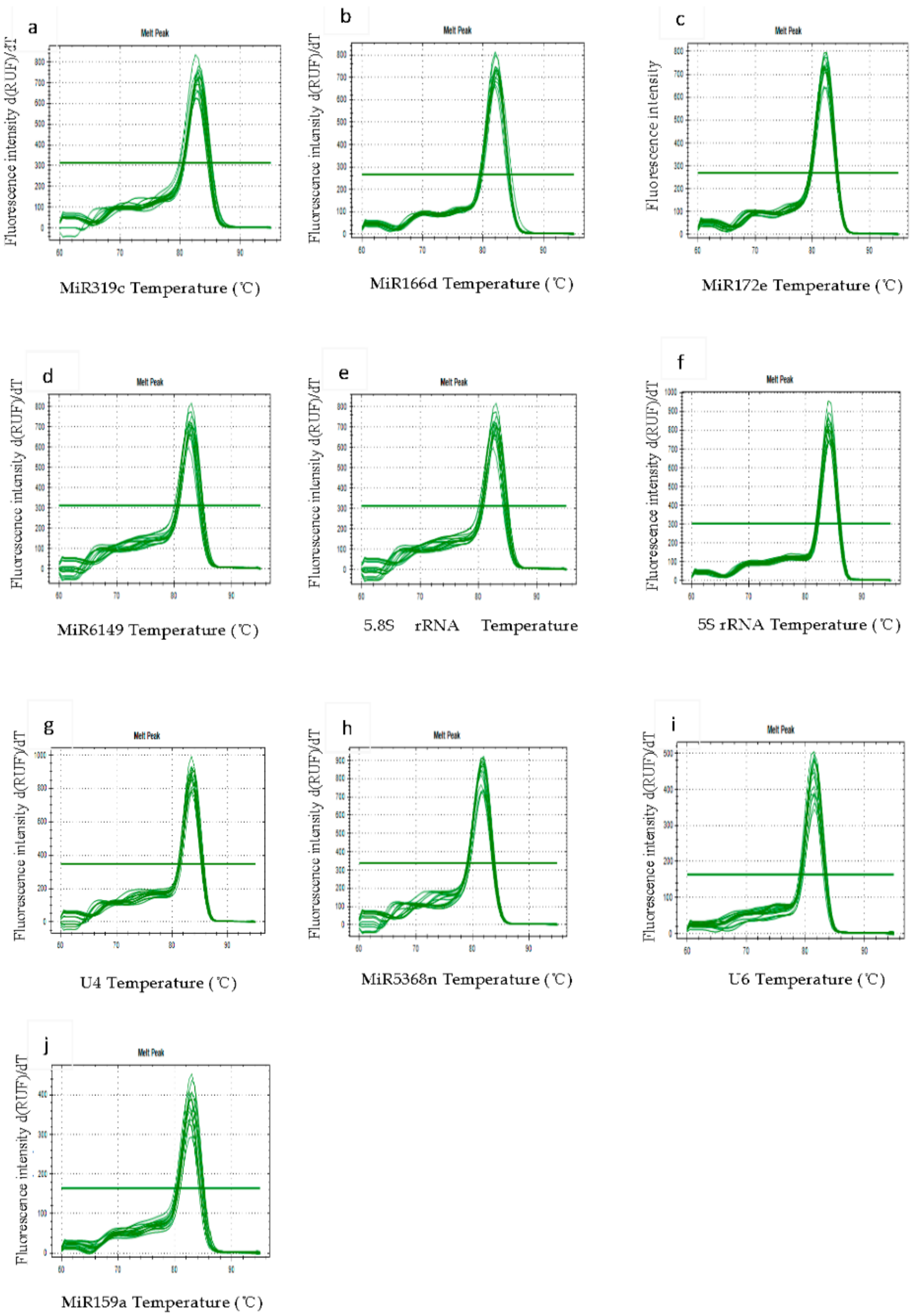

Figure S3. Melting curves of ten genes of qRT-PCR.
